# Clinical Efficacy and Toxicity of Anti-EGFR Therapy in Common Cancers

**DOI:** 10.1155/2009/567486

**Published:** 2009-05-06

**Authors:** Amir Harandi, Aisha S. Zaidi, Abigail M. Stocker, Damian A. Laber

**Affiliations:** Division of Hematology and Medical Oncology, J. G. Brown Cancer Center, University of Louisville, Louisville, KY 40202, USA

## Abstract

Epidermal growth factor receptor (EGFR) is a cell surface molecule and member of the ErbB family of receptor tyrosine kinases. Its activation leads to proliferation, antiapoptosis, and metastatic spread, making inhibition of this pathway a compelling target. In recent years, an increasing number of clinical trials in the management of solid malignancies have become available indicating the clinical efficacy of anti-EGFR monoclonal antibodies and oral small molecule tyrosine kinase inhibitors (TKIs). This review addresses frequently used EGFR inhibitors, summarizes clinical efficacy data of these new therapeutic agents, and discusses their associated toxicity and management.

## 1. Introduction

Epidermal growth factor receptor (EGFR), a member of the ErbB family of receptor tyrosine kinases, is a cell surface molecule whose activation leads to an intracellular signaling cascade affecting invasion, apoptosis, and angiogenesis [1, 2]. Members of the EFGR family receptors (erb1/EFGR, erb2/HER2, erb3/HER3, and erb4/HER4) are composed of extracellular ligand binding domains. When ligands bind to these domains, receptor dimerization and autophosphorylation of intracellular tyrosine kinase domains occur. Autophosphorylation activates the downstream signaling pathways ras, raf, mitogen-activated protein kinase (MAPK), phosphatidylinositol 3-kinase (Pl3K), Akt, and the signal transduction and activator of transcription (STAT) pathways. This downstream signaling leads to activation of cell growth, proliferation, and survival of cells [3, 4]. Binding of the EGFR by inhibitors leads to a disruption in proliferation resulting in apoptosis. Immunological effects, such as cell-dependent cytotoxicity (CDC) and antibody-dependent cell-mediated cytotoxicity (ADCC), also contribute to their mechanism of action [5].

Drugs targeting EGFR in malignancies were initially developed in the 1980s, which lead to the development of anti-EGFR monoclonal antibodies and small molecule EGFR tyrosine kinase inhibitors (TKIs) [6–9]. EGFR is overexpressed in many solid tumors and this over expression correlates to advanced stage and a worse prognosis [10]. In the last few years, numerous clinical trials have proven the clinical efficacy of EGFR-targeted therapies in the management of several cancers, including breast, colon, pancreas, head and neck, renal, gastrointestinal stromal tumors (GISTs), and lung carcinomas. Since these agents are now commonly used, clinical presentation of associated toxicities and their management are important to recognize. Therefore, this review discusses commonly used EFGR inhibitors currently approved by the US Food and Drug Administration (FDA). A summary of clinical data in support of these agents and commonly encountered toxicities and management are discussed.

## 2. Anti-EGFR Agents Efficacy

### 2.1. Erlotinib

Erlotinib is an oral agent that reversibly binds to the intracellular tyrosine kinase domain of the HER1/EGFR thus blocking phosphorylation and inhibiting signal transduction [11]. Initially studied in nonsmall cell lung cancer (NSCLC), phase II data showed a response rate (RR) of 12% in patients previously treated with platinum-based chemotherapy [12, 13]. The National Cancer Institute of Canada Clinical Trials Group (NCICCTG) then developed a phase III trial comparing erlotinib to placebo in patients with advanced NSCLC who had prior failure of first- or second-line chemotherapy. This study showed that erlotinib when compared to placebo had a higher overall (O)RR, median duration of response, progression-free survival (PFS), and overall survival (OS) (Table 1). There was also a greater reduction in cancer-related pain, cough, and dyspnea as well as improvement in physical function in those treated with erlotinib [14]. As a result, erlotinib is a useful treatment option presently utilized in the management of NSCLC. In another large phase III randomized trial of previously untreated advanced NSCLC, the combination of carboplatin and paclitaxel with or without erlotinib was evaluated. The results were not as favorable and showed no difference in ORR or OS [11]. EGFR gene mutations are being investigated as a predictor of efficacy with erlotinib in NSCLC. Recently presented at the American Society of Clinical Oncology (ASCO) Annual Meeting, a phase II trial of erlotinib in previously untreated NSCLC patients with mutations of the tyrosine kinase domain of EGFR was evaluated. In this trial, 37 of 297 tumors screened had mutations in the tyrosine kinase domain (25 with exon 19 deletion, 11 with L858R mutation). Responses occurred in 100% of exon 19 deletions and in 75% of those with the L858R mutation [15].

HER1/EGFRs are also overexpressed in pancreatic tumors conferring a worse prognosis. This led to an NCIC trial comparing gemcitabine in combination with erlotinib or placebo in patients with locally advanced or metastatic pancreatic adenocarcinomas. This trial showed a minimal but statistically significant increase in OS favoring the gemcitabine/erlotinib combination. Although statistically significant, the absolute increase in median survival was only 2 weeks [16].

### 2.2. Gefitinib

Gefitinib, an orally bioavailable EGFR TKI, was the first targeted drug to be approved for NSCLC. The Iressa Dose Evaluation in Adjuvant Lung Cancer (IDEAL1 and IDEAL2) trials were phase II nonrandomized studies investigating the efficacy of gefitinib monotherapy in NSCLC patients previously treated with a platinum agent [17, 18]. Based on objective responses, stable disease, and symptomatic improvement, gefitinib received accelerated approval by the FDA in 2003. In 2005, the Iressa Survival Evaluation in Lung Cancer (ISEL) trial, a phase III randomized study, evaluated gefitinib versus placebo in previously treated NSCLC [19]. Although there was a significantly higher response rate seen with gefitinib, the study did not show a significant difference in OS. As a result, the FDA restricted the use of gefitinib to patients enrolled in clinical trials or deriving benefit from ongoing treatment. Other randomized phase III trials, assessing gefitinib given concurrently with chemotherapy as well as gefitinib maintenance did not show improvements in OS [20–22]. Recently, the 33rd European Society for Medical Oncology Congress released results of a large-scale randomized phase III trial (IRESSA Pan-Asia study [IPASS]) [23]. In 1,217 patients, the study compared gefitinib monotherapy to carboplatin/paclitaxel (C/P) in chemonaive never- or light-exsmokers with advanced NSCLC with adenocarcinoma histology (Table 2). Gefitinib was superior in PFS, ORR, toxicity, and quality of life (QOL) compared to combination chemotherapy. However, OS and symptom improvement were similar between the two groups. PFS was longer for gefitinib than C/P in EGFR mutation positive patients and longer with C/P in mutation-negative patients.

### 2.3. Cetuximab

Cetuximab is a chimeric monoclonal IgG1 antibody that binds to the EGFR subsequently blocking phosphorylation of the receptor [24]. Cetuximab, initially approved for the treatment of metastatic colon cancer, has made a significant difference in the management of patients with this disease. A phase III trial comparing cetuximab monotherapy to best supportive care (BSC) showed improved OS and QOL in patients with colorectal cancer who had previously failed or had contraindications to fluoropyrimidine-, irinotecan-, and oxaliplatin-based therapies (Table 2) [25]. A subsequent randomized phase III trial compared cetuximab monotherapy to cetuximab plus irinotecan in refractory metastatic colorectal cancer (mCRC). This study was reserved for patients who had documented disease progression on a prestudy irinotecan regimen. The combination therapy arm showed significantly improved ORR, median time to progression (TTP), and disease control [26]. Another similar randomized phase III trial evaluated irinotecan monotherapy to cetuximab plus irinotecan in patients with mCRC previously failing oxaliplatin and/or a fluoropyrimidine who were irinotecan naïve. The combination therapy arm yielded improved ORR, PFS, and QOL, but similar OS to the cetuximab-only treated patients. This lack of OS difference may have been due to posttrial therapy since a large number of patients assigned to irinotecan eventually received cetuximab [27]. Most recently, the combination of irinotecan and 5-fluorouracil (FOLFIRI) with or without cetuximab in the first-line treatment of mCRC was evaluated. Cetuximab in combination with FOLFIRI significantly increased ORR and PFS [28].

The combination of EGFR and vascular endothelial growth factor-(VEGF-) targeted agents was also evaluated in a randomized phase III study of capecitabine/oxaliplatin (CapOx) plus bevacizumab with or without cetuximab in mCRC. Unfortunately, the combination chemotherapy, bevacizumab, and cetuximab resulted in a significant decrease in PFS compared to bevacizumab and CapOx alone with no difference in OS [29]. Therefore, it was concluded that cetuximab and bevacizumab should not be used concomitantly with chemotherapy.

At last year's ASCO annual meeting, data revealed that KRAS gene mutation conferred resistance to treatment with cetuximab and panitunimab. In contrast, “wild-type” or normal KRAS mutation status was found to be a predictive marker for cetuximab and panitumumab efficacy. In a retrospective analysis of 5-fluorouracil/leucovorin/oxaliplatin (FOLFOX) in combination with cetuximab, KRAS wild-type status was found to be associated with a significantly higher ORR and longer PFS when compared to mutant KRAS in EGFR-positive patients [29]. Similarly, when EGFR-positive patients with untreated mCRC were treated with FOLFIRI with or without cetuximab, KRAS mutational status was predictive of response; wild-type KRAS was associated with improved ORR and prolonged PFS. Based on these studies as well as data with panitumumab, KRAS testing and verification of wild-type status are now required before treatment with these agents in colorectal cancer [29–31].

EGFR is also upregulated in squamous cell carcinoma (SCC) of the head and neck. The use of cetuximab to treat this disease has significantly benefited these patients. Initial phase II data revealed activity of single agent cetuximab in recurrent and/or metastatic SCC of the head and neck in those failing to respond to platinum-based therapy. As a single agent, the RR was 13% with a disease control rate of 46% [32]. The combination of cetuximab with chemotherapy has also been found to be effective. Platinum-based chemotherapy, fluorouracil, with or without cetuximab as first-line treatment of metastatic or recurrent SCC of the head and neck showed that the cetuximab combination yielded a higher ORR and OS [33]. A similar phase III trial was conducted addressing PFS with cisplatin monotherapy versus cisplatin with cetuximab in patients with recurrent and/or metastatic SCC of the head and neck. This included patients with documented progression during prior cisplatin therapy. This study did not show a significant difference in PFS or OS; however, there was a significant difference in RR favoring the cetuximab/cisplatin arm [34]. Cetuximab with radiotherapy versus radiotherapy alone was also studied in a separate phase III trial. The addition of cetuximab to radiotherapy significantly prolonged PFS, median OS, and duration of locoregional control in patients with locoregionally advanced head and neck cancer [35].

In pancreatic adenocarcinoma, trials with cetuximab have not shown significant clinical benefit. Cetuximab with gemcitabine or gemcitabine alone was evaluated in a large multi-institutional phase III trial in pancreatic adenocarcinoma. The addition of cetuximab did not significantly improve ORR, PFS, or OS [36–38].

At the plenary session of ASCO 2008, data was released on the treatment of NSCLC with cetuximab in combination with cisplatin/vinorelbine (CV) compared to CV alone. Only patients with EGFR detectable by immunohistochemistry (IHC) were randomized. Cetuximab plus CV demonstrated an OS advantage. A modest survival benefit of one-to-two month(s) was seen depending on histology. This was the first trial to demonstrate an OS advantage of an EGFR-targeted agent in combination with platinum-based chemotherapy in NSCLC [39].

EGFR gene copy number detected by fluorescent in situ hybridization (FISH) has been shown to be useful in selecting NSCLC patients for treatment with cetuximab. Patients with advanced-stage NSCLC were enrolled into a phase II trial evaluating sequential or concurrent chemotherapy (carboplatin plus paclitaxel) with cetuximab. The ORR, disease control rate, PFS, and OS were significantly higher in the FISH-positive versus FISH-negative patients [40]. Further investigation on the accuracy of FISH-positive EGFR status is needed to evaluate its prognostic value in NSCLC.

### 2.4. Panitumumab

In contrast to cetuximab, panitumumab is the first fully human EGFR monoclonal antibody. It is an immunoglobulin (Ig)G2 antibody that binds to the extracellular portion of the EGFR thus inhibiting phosphorylation and activation of the intracellular kinases [41]. Efficacy of panitumumab has been evaluated in EGFR-expressing metastatic colorectal adenocarcinomas with disease progression following oxaliplatin, irinotecan, and fluropyrimidine-containing chemotherapy regimens. An initial phase II multicenter trial included patients with progressive mCRC treated with panitumumab monotherapy; patients were stratified into two groups based on EGFR staining intensity. As a single agent, panitumumab response and disease stabilization were seen irrespective of EGFR staining intensity [42]. This led to a phase III trial comparing panitumumab monotherapy to BSC alone. Efficacy was evaluated in patients with 1% or greater EGFR tumor staining by IHC and disease progression while on or within 6 months of the most recent chemotherapy. Panitumumab yielded a significant reduction in PFS when compared to BSC; however, there was no significant difference in OS (Table 3) [43]. Patients in the BSC arm were subsequently allowed to crossover to the panitumumab arm if disease progression was documented during the study. The crossover patient population yielded comparable results with prolonged PFS after panitumumab treatment [44]. This has led to the approval of panitumumab for treatment of EGFR-positive, metastatic colorectal carcinoma with disease progression following chemotherapy [41]. As mentioned earlier, wild-type KRAS is required for panitumumab efficacy in patients with mCRC [31].

The combination of EGFR- and VEGF-targeting antibodies was also found to lack benefit in the case of panitumumab. In the Panitumumab Advanced Colorectal Cancer Evaluation (PACCE) study, patients with untreated mCRC were randomized to FOLFOX or FOLFIRI based on investigator or patient choice. This combination was given with panitumumab plus bevacizumab or bevacizumab alone. The combination of FOLFOX/panitumumab/bevacizumab resulted in higher mortality compared to FOLFOX/bevacizumab alone. The primary endpoint of median PFS was also shorter in the panitumumab arm. Based on the results of the interim analysis, the study was stopped and panitumumab was discontinued in both the FOLFOX and FOLFIRI arms [45]. Similar to cetuximab, this trial with panitumumab argues against the combined use of these agents with bevacizumab in mCRC.

### 2.5. Sorafenib

Sorafenib is a novel multikinase inhibitor with antiangiogenic and proapoptotic activity targeting EGFR as well as multiple kinases including Raff/MAPK-ERK kinase, VEGFR-2, VEGFR-3, and PDGFR-*β* [39]. It is approved for use in the treatment of renal cell carcinoma (RCC) and hepatocellular carcinoma (HCC).

RCC is characterized by the loss of the von hippel landau (VHL) gene, which leads to dysregulation of the VEGFR, PDGFR-*β*, transforming growth factor-alpha (TGF-) *α*, EGFR, and Raf pathways promoting angiogenesis, lymphangiogenesis, tumor cell growth, and survival. Furthermore, RCC frequently displays EGFR immunoreactivity. Membranous and/or cytoplasmic EGFR immunostaining in RCC was present in 123 of 132 (93%) primary and 49 of 53 (92%) metastatic samples with extensive immunoreactivity present in 83% of primary and 74% of metastatic tumors [46].

In previously treated patients with metastatic RCC, the activity of sorafenib was demonstrated in two randomized trials. In the largest of these studies, a randomized phase III trial of metastatic cytokine refractory RCC, significant response and improvement in PFS was demonstrated (Table 4). At ASCO 2007, a final analysis of survival was presented. There was no statistically significant improvement in OS; survival benefit was likely obscured since one half of the patients originally assigned to placebo had switched to sorafenib [47].

EGFR is frequently expressed in human hepatoma cells; in fact, EGF is one of the mitogens required for the growth of hepatoma cells. At the ASCO meeting in 2007, data was released showing the efficacy of sorafenib in HCC. The Sorafenib HCC Assessment Randomized Protocol (SHARP) Trial was a large phase III double-blind placebo-controlled study evaluating the efficacy of sorafenib versus BSC in patients with advanced HCC who had not received previous chemotherapy. Patients receiving sorafenib had a three-month median survival benefit compared to placebo. Importantly, sorafenib was the first active treatment that has been proven to confer a survival benefit and to show promise as a standard treatment for advanced HCC [48]. In another phase III randomized trial of sorafenib in Asia-Pacific patients with HCC, results mirrored those of the SHARP trial. Despite Asia-Pacific patients having more advanced disease based on the Eastern Cooperative Oncology Group performance status (ECOG PS), a significant OS advantage with sorafenib was confirmed [49]. A North American phase II randomized trial of doxorubicin with sorafenib versus doxorubicin with placebo in 96 Child-Pugh A patients was published in abstract form and presented at the 2007 European Cancer Organization Conference (ECCO). Median TTP was two months longer in the combination arm, but did not reach statistical significance. However, OS was significantly longer for the combination of sorafenib/doxorubicin compared to the doxorubicin only arm (13.7 vs. 6.5 mo) [50].

Sorafenib has also recently demonstrated significant activity in the treatment of iodine-refractory thyroid carcinoma. In a phase II trial of 30 subjects, most of the patients (80%) showed a clinical benefit from this agent. Ninety-five percent of individuals with available thyroglobulin levels showed a rapid response in thyroglobulin levels with a mean decrease of 70%. These results represent a significant advance in both response and PFS over studies in the past utilizing chemotherapy [51].

### 2.6. Sunitinib

Like sorafenib, sunitinib is an oral small molecule TKI that inhibits cellular signaling by targeting EGFR, VEGFR, PDGFR-*β*, fetal liver tyrosine kinase receptor (FLT-3), and c-Kit, a stem cell factor receptor [52]. This ultimately targets both angiogenesis and tumor cell proliferation causing tumor shrinkage and cell death. Sunitinib is currently approved for the treatment of RCC as well as gastrointestinal stromal tumors GISTs. Like RCC, EGFR expression in GISTs had been validated in a recent article in which tissue microarray samples of 33 GISTs were surveyed by IHC. EGFR expression was identified in 8 of those samples [53].

The antitumor activity of sunitinib was initially shown in two phase II trials of metastatic RCC patients who had failed previous cytokine therapy [54, 55]. This led to a large phase III trial comparing sunitinib to interferon-alpha (IFN-*α*) as first-line therapy (Table 5). Sunitinib showed superior activity in ORR and in PFS, including patients with good, intermediate, and poor risk features. Furthermore, OS was significantly longer in the sunitinib arm, despite significant patient crossover from the IFN-*α* arm to sunitinib [56]. Sunitinib was also found to be superior in QOL compared to IFN-*α* [57].

Sunitinib has also shown significant activity in metastatic and/or unresectable GIST following imatinib failure. In a phase III randomized trial comparing sunitinib to placebo in imatinib refractory GIST patients, time to tumor progression and PFS was 4-fold longer in patients on sunitinib compared to placebo; partial response (PR) and stable disease (SD) were also significantly longer in the sunitinib arm. Patients in the placebo arm were subsequently allowed to crossover to the Sunitinib arm if disease progression was documented during the study. The crossover patient population yielded comparable results. Despite the crossover, OS favored patients initially treated with sunitinib [58].

### 2.7. Lapatinib

Activation and overexpression of oncogenes encoding trans-membrane receptor tyrosine kinases of the EGFR family, including EGFR (ErbB1) and HER2/neu (ErbB2), play an important role in the development of breast cancer [58]. Lapatinib is an orally active 4-anilinoquinazoline TKI of both HER2/neu (ErbB2) and EGFR (ErbB1). It inhibits the autophosphorylation sites on the receptors, thereby blocking the downstream signaling pathways of HER2 and EGFR.

Lapatinib has shown activity for the treatment of advanced HER2/neu positive metastatic breast cancer refractory to trastuzumab (Table 6). Unlike trastuzumab, lapatinib seems to have activity against brain metastases [59–61]. Primary and secondary resistances have been seen in patients with HER2-positive breast cancers who had been treated with trastuzumab both in the metastatic and adjuvant settings [53, 62–65]. Potential mechanisms of resistance may be related to signaling through other receptors such as EGFR or IGFR-1 [66]. Lapatinib, being a small molecule TKI, interacts with intercellular domains and does not require full receptor activity. In a phase III study of HER2-positive advanced or metastatic breast cancer refractory to anthracyclines, taxanes, and trastuzumab, lapatinib plus capecitabine showed a significant advantage over capecitabine monotherapy with respect to ORR and PFS with a nonsignificant trend toward longer OS [67]. In another phase III randomized trial, the combination of paclitaxel with lapatinib compared to paclitaxel monotherapy in patients with HER2-positive cancer was evaluated. Patients in this trial were not treated with prior trastuzumab. The study showed a statistically significant advantage in ORR and TTP, but not in OS. Enrollment for this study came from countries with limited HER2 testing. Only 91 of 580 patients were HER2-positive on central testing, with retrospective analysis revealing benefit limited to FISH-positive or IHC 3+ tumors [68].

## 3. Anti-EGFR Agent-Associated Toxicity

EGFR is expressed on nearly all normal cells, particularly those of epithelial origin such as skin, liver, and gastrointestinal tract, but not on hematopoietic cells [69]. As a consequence, the most commonly encountered toxic effects from these agents are rash and diarrhea. Along with other toxicities, recognition and management of associated adverse effects with anti-EGFR agents will result in improved clinical outcomes, patient compliance, and QOL.

### 3.1. Skin Toxicity

When EGF was initially discovered, it was named for its ability to increase growth and keratinization of skin epithelium [69]. EGFR was found to be expressed in the human skin within keratinocytes, the follicular epithelium, sweat and sebaceous glands, and in capillaries of the dermis [70–73]. For this reason, the most common toxicity of EGFR-targeted agents involves the skin and adnexal structures resulting in a rash and less commonly nail toxicity.

EGFR is expressed on hair follicles and sebaceous glands and the binding of this receptor by inhibitors leads to a disruption in proliferation, resulting in an immunological reaction with skin inflammation, folliculitis, and rash [72, 73]. The most commonly seen skin reaction with EFGR inhibitors is a follicular acneiform eruption, also termed acne-like rash or folliculitis. EGFR-associated rash differs from acne in that there are no comedones or blackheads. The incidence of an acneiform-like skin rash has been reported to occur in about 85% of cetuximab-treated patients [74, 75]. Symptoms typically appear within two weeks after starting treatment. This is mainly located on the face (nose, cheeks, nasolabial folds, chin, forehead, and in a perioral distribution). Other locations include the shoulders and upper part of the back and chest. The rash tends to improve over time even with continued use and does resolve fully after cessation of therapy. In 35% of patients, dry itchy skin of the arms and legs can occur, which can potentially become secondarily infected by *Staphylococcus aureus* or *Herpes simplex* infection [76].

There have been numerous studies showing a direct correlation between the severity of a rash with response and OS. In fact, the greatest benefit in survival of cetuximab-treated patients is seen in those with a grade 3 rash [27, 75, 76]. The degree of skin toxicity has been classified by The National Cancer Institute Common Toxicity Criteria version 3.0 (Table 7). Prospective trials are needed to further investigate the correlation between anti-EGFR therapy and rash to elucidate the validity and clinical implications of this association.

Recommendations in the management of EGFR-associated rash have been limited by the lack of clinical trials evaluating rash therapies (Table 8). This has led to treatment recommendations based on the clinical experience of dermatologists and oncologists familiar with EGFR-associated rash. In general, preventive measures are essential and include avoidance of soaps, limiting shower time, use of lukewarm water, and liberal use of skin moisturizers and emollients. Beneficial topical treatment approaches for a grade 1 rash include the use of antibiotic agents (metronidazole, erythromycin, and clindamycin lotion) as well as local corticosteroid cream if an extensive inflammatory component is present. For grade 2 reactions, anti-inflammatory oral antibiotics (minocycline or doxycycline) should be used since secondary infections are common. In the event of a grade 3 rash, oral corticosteroids and antibiotics should be utilized and EGFR-targeted therapy should be held until the acute inflammatory phase has resolved [77, 78].

Rash and hand-foot syndrome, characterized by redness, ulceration, and dysesthesia of the palms and soles, are the most common adverse events associated with sorafenib and sunitinib. Hand-foot syndrome associated with these agents occurs in 20–30% of patients, with less than 10% experiencing grade 3 or higher toxicity. It rapidly resolves with drug discontinuation and topical emollients and moisturizers are used to prevent and diminish toxicity. Other associated adverse events seen with sorafenib and sunitinib include diarrhea, hypertension, fatigue, and hematologic cytopenias [79, 80].

Anti-EGFR therapy can also result in nail toxicity, which can occur in 10–15% of patients after 4–8 weeks of therapy. It can progress into a paronychia like cracking reaction, a painful and difficult to treat side effect [74]. Some individuals require several months for complete healing after cessation of therapy [71]. Hair disorders are also commonly seen with these agents. Since EGFR signaling plays a vital role in the initiation of hair growth, interruption of EGFR signaling can result in disorganized hair follicles leading to follicular necrosis and alopecia [81, 82].

Nimotuzumab, which is marketed under the name of BIOMAbegfr, is a recombinant humanized IgG1 monoclonal antibody targeting EGFR. It has been approved for SCC of the head and neck and glioma in a number of countries. In the clinical trials thus far, nimotuzumab has not been associated with skin toxicity. Further clinical trials in different cancers are currently ongoing [83].

### 3.2. Gastrointestinal Toxicity

The use of oral anti-EGFR TKIs has been found to be strongly associated with gastrointestinal toxicities including diarrhea and hepatotoxicity. The pathophysiology of anti-EGFR-induced diarrhea is thought to result from excessive chloride secretion inducing a secretory diarrhea [84]. In large randomized trials, oral TKIs erlotinib and lapatinib have been found to cause diarrhea in 40–60% of patients with approximately 10% experiencing grade 3 or 4 toxicity [11, 14]. The reported incidence of diarrhea associated with sorafenib and sunitinib has been 20–40% [79, 81]. Diarrhea induced by oral TKIs can be managed by lowering the dose and rarely involves treatment interruption. Loperamide is a useful therapy decreasing intestinal motility.

Hepatic toxicity with asymptomatic elevations of transaminases and hyperbilirubinemia is commonly associated with oral TKIs. The mechanism of action is thought to be direct targeting of hepatocytes that overexpress EGFR with potential induction of chronic hepatitis with active necrosis. The overall incidence of hepatotoxicity with oral anti-EGFR TKIs has been reported at around 10% (2% grade 2 or 3) [11, 14, 79, 80]. These compounds can be continued with mild hyperbilirubinemia and should be discontinued with grade 3 or 4 toxicity. Concurrent TKIs and hepatotoxic drugs should be used with caution.

### 3.3. Pulmonary Toxicity

Interstitial lung disease related to gefitinib therapy has been well reported, with a worldwide incidence estimated at 1% [85, 86]. In a small series from Japan, the incidence of ILD in 112 patients receiving gefitinib was estimated at 5.4% [87]. Four deaths occurred, all being in current or former smokers, with pre-existing pulmonary fibrosis being a significant risk factor. The adverse pulmonary effects of erlotinib are less well known, but cases of fatal ILD have been reported [88, 89]. A phase 3b trial has been initiated to examine the efficacy and safety of erlotinib in advanced NSCLC with disease progression after chemotherapy. From a total of 229 patients, one (0.4%) interstitial lung disease-like event was reported [90].

### 3.4. Cardiac Toxicity

Cardiac toxicity with anti-EGFR TKIs has been reported. It is clear that cardiotoxicity with TKIs is not a “class effect,” since it does not occur with all known agents. Sunitinib caused a decline in left ventricular ejection fraction (LVEF) below 50% in 11% of the patients [91]. In a phase III trial comparing sunitinib to IFN-*α*, 10% of patients had declines in LVEF after a median duration treatment of 6 months [56]. Sorafenib has induced acute coronary syndromes, including myocardial infarction. In the RCC study comparing sorafenib to placebo, 2.9% of sorafenib-treated patients had a myocardial infarction, compared to 0.4% in placebo-treated patients [47]. The cardiac toxicity of lapatinib was analyzed in 3.558 patients treated in 18 phase I–III clinical trials; 598 received prior anthracyclines and 759 had been given trastuzumab in the past [92]. Lapatinib was associated with a decline in LVEF in 1.6% of patients (58 of 3,558). The mean LVEF decrease was 18.7% and was mostly asymptomatic (1.4% asymptomatic and 0.2% symptomatic). Of the seven with symptomatic LVEF decrease, cardiotoxicity resolved in all but one patient.

### 3.5. Allergic Reactions

Allergic and anaphylactoid reactions are associated with cetuximab and, less often, panitumumab administration [26, 75]. Severe reactions are observed in approximately 3% of patients following cetuximab administration, with a fatal outcome in 0.1% of patients [93, 94]. Up to 90% of severe reactions associated with cetuximab occur within the first few minutes of the first dose [95]. The decision to rechallenge or discontinue treatment after a reaction occurs depends on the severity of the reaction. In the case of anaphylactic reactions, further therapy with cetuximab is contraindicated. Mild-to-moderate hypersensitivity reactions can be managed by temporary infusion interruption and resuming at a slower infusion rate. Management of severe reactions must include immediate interruption and treatment with epinephrine. Corticosteroids, antihistamines, bronchodilators, and oxygen also might be required.

Hypersentivity reactions to cetuximab might correlate with the development of specific antihuman IgE antibodies [96]. On the contrary, no antihuman antibodies have been detected with panitumumab. The incidence of hypersensitivity reactions with panitumumab in multiple trials has been approximately 3%, with severe reactions accounting for approximately 1% [97]. The successful use of panitumumab after severe hypersensitivity reactions to cetuximab has been reported, but requires further investigation [98].

## 4. Conclusions

Therapeutic agents in clinical practice targeting the EGFR pathway have made great advances in the treatment of malignancy. EGFR activation is associated with proliferation, antiapoptosis, and metastatic spread, making this pathway a compelling target. Numerous large clinical trials have shown clinical evidence of anticancer activity with these new agents resulting in improved tumor response and patients' survival. Several other anti-EGFR agents are in development, giving hope to future advances in therapy. An awareness and proper management of associated toxicities can increase patient compliance, QOL, and overall treatment success. Ongoing and future research will expand the applications of anti-EGFR therapy, elucidate optimal combinations and sequences, discover pathways of resistance, and continue to benefit cancer patients.

## Figures and Tables

**Table 1:** Selected clinical trials of erlotinib. *NSCLC,* Non-small cell lung cancer; *OS,* overall survival; *ORR,* overall response rate.

Malignancy	Regimen	Number of patients	Results	Comments
NSCLC	Erlotinib vs. placebo [14]	731 pts Stage IIIB/IV NSCLC after failure with first-line or second-line chemotherapy	Erlotinib: ORR (8.9%) OS (6.7 mo) Placebo: ORR (<1%) OS (4.7 mo)	Significant improvement in OS (*P* < .001)

NSCLC	Carboplatin, Paclitaxel +/− Erlotinib [11]	1059 pts Previously untreated stage IIIB/IV NSCLC	Erolotinib: ORR (21.5%) OS (10.6 mo) Placebo: ORR (19.3%) OS (10.5 mo)	No difference in ORR or OS with the combination of Erlotinib and chemotherapy

Pancreatic cancer	Gemcitibine, Erlotinib vs. placebo [15]	569 pts Unresectable, locally advanced or metastatic pancreatic cancer	Erlotinib: OS (6.2 mo) Placebo: OS (5.9 mo)	One year survival was greater with erlotinib plus gemcitabine (23% vs. 17%; *P* = .023)

**Table 2:** Selected clinical trials of gefitinib. *NSCLC,* Non-small cell lung cancer; *OS,* overall survival; *ORR,* overall response rate; C/P, carboplatin/paclitaxel; *PFS,* progression free survival; *EGFR,* epidermal growth factor receptor.

Malignancy	Regimen	Number of patients	Results	Comments
NSCLC	Gefitinib vs. placebo [19]	1692 pts Second-line or third-line treatment for patients with locally advanced or metastatic NSCLC	Gefitinib: OS (5.6 mo) Placebo: OS (5.1 mo)	No significant improvement in OS (*P* = .087) Subgroup analysis showed significantly longer survival in never-smokers and Asian patients

NSCLC	Gefitinib vs. Carboplatin/paclitaxel (C/P) [23]	1,217 pts Previously untreated stage IIIB/IV NSCLC, never- or light ex-smokers, adenocarcinoma histology	Gefitinib: ORR (43%) OS (18.6 mo) C/P: ORR (32%) OS (17.3 mo) *P* = .0001	No OS difference PFS longer for gefitinib than C/P in EFGR mutation positive patients (*P* < .0001) PFS longer with C/P in mutation negative patients (*P* < .0001)

**Table 3:** Selected clinical trials of cetuximab. *EGFR,* epidermal growth factor receptor; *BSC,* best supportive care; *OS,* overall survival; *PFS,* progression free survival; *ORR,* overall response rate; *mCRC,* metastatic colorectal cancer; *FOLFIRI,* 5 Flourouracil/Folinic Acid and Irinotecan; *CapOx*, capecitabine/oxaliplatin; *SCC,* squamous cell carcinoma; IHC, Immunohistochemistry; *CR*, complete response; *PR,* partial response; *FISH*, fluorescent in situ hybridization.

Malignancy	Regimen	Number of patients	Results	Comments
mCRC	Cetuximab vs. BSC [17]	572 pts IHC EGFR+ mCRC Previously treated with chemotherapy	Cetuximab: PR (8%) SD (31.4%) BSC: PR (0%) SD (10.9%)	Cetuximab was associated with a significant improvement in OS (*P* < .001) Cetuximab: OS (6.1 mo), BSC: OS (4.6 mo)

mCRC	Cetuximab, Irinotecan vs. Cetuximab monotherapy [18]	329 pts mCRC with progression after Irinotecan-based chemotherapy	Cetuximab, Irinotecan: ORR (22.9%) Cetuximab: ORR (10.8%)	No difference in OS Median time to progression: Cetuximab, Irinotecan (4.1 mo), Cetuximab (1.5 mo)

mCRC	Cetuximab, Irinotecan vs. Irinotecan [19]	1298 pts EGFR+ mCRC	Cetuximab, Irinotecan: ORR (16.4%) PFS (4.0 mo) Cetuximab: ORR (4.2%) PFS (2.6 mo)	No significant difference in OS, but large number of pts receiving Irinotecan eventually got cetuximab

mCRC	FOLFIRI +/− Cetuximab [20]	1,217 pts EGFR+ mCRC First-line treatment	FOLFIRI + Cetuximab: PFS (8.9 mo) ORR (46.9%) FOLFIRI alone: PFS (8 mo) ORR (38.7%)	15% relative risk reduction of progression

mCRC	CapOx, bevacizumab +/− Cetuximab [21]	775 pts Previously untreated mCRC	CapOx, bevacizumab: ORR (40.6%) PFS (10.7 mo) Cetuximab arm: ORR (43.9%) PFS (9.8 mo)	Cetuximab combination was worse in PFS No difference in OS

mCRC	FOLFOX +/− Cetuximab [22]	337 pts 134 pts wild-type KRAS 99 pts mutant KRAS	Wild-type KRAS response with FOLFOX + Cetuximab (ORR 61%, PFS 7.7 mo) Mutant KRAS response with FOLFOX + Cetuximab (ORR 33%, PFS 5.5 mo)	Cetuximab only benefits patients with wild-type KRAS (HR 0.448, *P* = .0009)

mCRC	FOLFIRI +/− Cetuximab [23]	1,217 pts 348 pts wild-type KRAS 192 pts mutant KRAS	Wild-type KRAS response with FOLFIRI + Cetuximab (ORR 59%, PFS 9.9 mo) Mutant KRAS response with FOLFIRI + Cetuximab (ORR 36%, PFS 7.6 mo)	Cetuximab only benefits patients with wild-type KRAS and reduced risk for disease progression by 32% (*P* = .017)

SCC of the Head and Neck	Platinum (cisplatin or carboplatin), fluorouracil +/− Cetuximab [25]	442 pts Untreated recurrent or metastatic SCC of the head and neck	Platinum, fluorouracil, Cetuximab: ORR (36%) PFS (5.6 mo) Platinum, fluorouracil: ORR (20%) PFS (3.3 mo)	Median OS was significantly improved in the Cetuximab arm (10.1 mo vs. 7.4 mo), *P* = .04

SCC of the Head and Neck	Cisplatin, Cetuximab vs. Cisplatin [26]	117 pts Recurrent/metastatic SCC of the head and neck	Cisplatin, Cetuximab: ORR (26%) Cisplatin ORR (10%)	No significant improvement in OS or PFS Enhanced response for patients with EGFR staining less than 80% by IHC

SCC of the Head and Neck	Radiation, Cetuximab vs. Radiation alone [27]	424 pts Locoregionally advanced SCC of the head and neck	Radiation, Cetuximab: PFS (17.1 mo) OS (49 mo) Radiation alone: PFS (12.4 mo) OS (29.3 mo)	OS benefit favoring Cetuximab arm (*P* = .03) Incidence in grade 3 or higher side effects, including mucositis, did not differ significantly between the groups

Pancreatic cancer	Cetuximab, Gemcitabine vs. Gemcitabine alone [30]	735 pts	Cetuximab, Gemcitabine: ORR (14%) PFS (3.5 mo) OS (6.4 mo) Gemcitabine alone: ORR (12%) PFS (3 mo) OS (5.9 mo)	The addition of Cetuximab did not significantly improve ORR, PFS, or OS

NSCLC	Cisplatin, Vinorelbine +/− Cetuximab [31]	1,125 pts Only pts with EGFR detected by IHC were randomized	Cisplatin, Vinorelbine, Cetuximab: Median OS (11.3 mo) Cisplatin, Vinorelbine: Median OS (10.1 mo)	OS significantly improved in Cetuximab arm (*P* = .04)

NSCLC	Sequential or concurrent carboplatin and paclitaxel with cetuximab [32]	229 pts EGFR by FISH assessable in 76 pts (positive in 59%)	FISH-positive: CR/PR (81%) Median PFS (6 mo) FISH-negative: CR/PR (55%) Median PFS (3 mo)	Median OS superior in FISH-positive (15 mo vs. 7 mo), *P* = .04

**Table 4:** Selected clinical trials of panitumumab. *mCRC,* metastatic colorectal cancer; *BSC,* best supportive care; *OS,* overall survival; *PFS,* progression free survival; *ORR,* overall response rate; *SD,* stable disease; *FOLFOX,* 5 Flourouracil/Folinic Acid and oxaliplatin; *FOLFIRI,* 5 Flourouracil/Folinic Acid and Irinotecan.

Malignancy	Regimen	Number of patients	Results	Comments
mCRC	Panitumumab vs. BSC [35]	463 pts Pts with progression after standard chemotherapy	Panitumumab: ORR (10%) PFS (13.8 weeks) BSC: ORR (0%) PFS (8.5 weeks)	No significant improvement in OS

mCRC	Panitumumab monotherapy after disease progression with BSC [36]	176 pts Pts with progression of disease in BSC arm of Panitumumab vs. BSC trial [35]	Panitumumab: ORR (11.6%) SD (33%) Median PFS of 9.4 weeks	Results comparable to initial study

mCRC	FOLFOX or FOLFIRI with Bevacizumab +/− Panitumumab [37]	823 pts	FOLFOX, Bevacizumab, Panitumumab: Median PFS (9.5 mo) OS (19.3 mo) FOLFOX, Bevacizumab: Median PFS (11 mo) OS (20.6 mo)	Panitumumab in combination with FOLFOX and bevacizumab was associated with a shorter PFS and increased toxicity

**Table 5:** Selected clinical trials of sorafenib. *PFS*, progression free survival; *OS,* overall survival; *PR*, partial response; *CR*, complete response; *HCC*, hepatocellular carcinoma; *RCC*, renal cell carcinoma; *TBRR,* Tumor burden reduction rate.

Malignancy	Regimen	Number of patients	Response rate	Comments
RCC	Sorafenib vs. placebo [41]	903 Resistant to standard therapy	Sorafenib: Median PFS (5.5 mo) PR (10%) Placebo: Median PFS (2.8 mo) *P* < 0.01 PR (2%)	The OS showed reduced risk of death compared with placebo but the results were not statistically significant

HCC	Sorafenib vs. placebo [42]	602 pts No previous therapy	Sorafenib: Median OS (10.7 mo) Placebo: Median OS (7.9 mo)	The median OS was significantly longer in patients who received Sorafenib HR (0.69), *P* = .0006

Metastatic thyroid carcinoma	Sorafenib monotherapy [45]	36 pts Metastatic, iodine-refractory thyroid carcinoma	PR in 7 pts (21%) SD in 20 pts (59%)	Significant anti-tumor activity with overall clinical benefit rate (PR + SD) of 80%

**Table 6:** Selected clinical trials of sunitinib. *GIST,* gastrointestinal stromal tumor; *PFS*, progression free survival; *OS,* overall survival; *PR*, partial response; *CR*, complete response; *TTP,* time to progression; *m RCC*, metastatic renal cell carcinoma; *QOL,* quality of life.

Malignancy	Regimen	Number of patients	Response rate	Comments
RCC	Interferon vs. Sunitinib [48]	750 pts Previously untreated mRCC	Sunitinib: Median PFS (11 mo) ORR (31%) OS (26.4 mo), *P* = 0.051 Interferon: Median PFS (5 mo) ORR (6%) OS (21.8 mo)	Sunitinib provides superior QOL compared with IFN-*α* in mRCC patients.

GIST	Sunitinib vs. placebo [50]	312 pts After progression or intolerance to imatinib	Sunitinib: TTP(6.3 mo) Placebo: TTP(1.5 month)	Sunitinib significantly improved TTP with a 67% reduced risk of progression.

**Table 7:** Selected clinical trials of lapatinib. *MBC,* metastatic breast cancer; *PFS*, progression free survival; *OS,* overall survival; *TTP,* time to progression.

Malignancy	Regimen	Number of patients	Response rate	Comments
MBC	Capecitibine +/− Lapatinib [59]	324 pts HER2-positive MBC that had progressed with chemotherapy (anthracycline, a taxane, and trastuzumab)	Capecitabine, Lapatinib: TTP (6.2 mo) ORR (24%) Capecitabine monotherapy: TTP (4.3 mo) ORR (14%)	Non-significant trend toward improved OS favoring lapatinib Fewer pts in the lapatinib arm developed brain metastases as the first site of progression (13 vs. 4%)

MBC	Lapatinib, Paclitaxel vs. Paclitaxel monotherapy [60]	580 pts 55% received prior chemotherapy or hormonal therapy No pts received prior traztuzumab	Lapatinib, Paclitaxel: ORR (60%) Median TTP (8 mo) Paclitaxel monotherapy: ORR (36%) Median TTP (6 mo)	Improved clinical outcome was seen with the combination without a significant change in side effect profile No difference in OS, but majority of pts were not properly tested for HER2

**Table 8:** Management of anti-EGFR-associated rash and common terminology criteria for adverse events v3.0 (CTCAE), National Cancer Institute.

CTC Grade	Rash	Management
1	Macular or papular eruption or erythema Asymptomatic	Topical antibiotic agents (metronidazole, erythromycin, and clindamycin lotion) Corticosteroid cream if an extensive inflammatory component exists

2	Macular or papular eruption or erythema Symptomatic covering <50% of body	Anti-inflammatory oral antibiotics (minocycline or doxycycline) Corticosteroid cream if an extensive inflammatory component exists

3	Macular or papular eruption or erythema Symptomatic covering >50% of body	Anti-inflammatory oral antibiotics (minocycline or doxycycline) Oral corticosteroids EGFR therapy should be held until the acute inflammatory phase has resolved

4	Generalized exfoliative, ulcerative, or bullouis dermatitis	Anti-inflammatory oral antibiotics (minocycline or doxycycline) Oral corticosteroids (Medrol-dose pack) EGFR therapy should be held until the acute inflammatory phase has resolved
